# A Network-Based Approach for Improving Annotation of Transcription Factor Functions and Binding Sites in *Arabidopsis thaliana*

**DOI:** 10.3390/genes14020282

**Published:** 2023-01-21

**Authors:** Tanzira Najnin, Sakhawat Hossain Saimon, Garry Sunter, Jianhua Ruan

**Affiliations:** 1 Department of Computer Science, The University of Texas at San Antonio, San Antonio, TX 78249, USA; 2 Department of Biological Sciences, Northern Illinois University, DeKalb, IL 60115, USA

**Keywords:** transcriptional regulatory network, network topology, gene expression pattern, gene ontology, functional annotation, motif discovery

## Abstract

Transcription factors are an integral component of the cellular machinery responsible for regulating many biological processes, and they recognize distinct DNA sequence patterns as well as internal/external signals to mediate target gene expression. The functional roles of an individual transcription factor can be traced back to the functions of its target genes. While such functional associations can be inferred through the use of binding evidence from high-throughput sequencing technologies available today, including chromatin immunoprecipitation sequencing, such experiments can be resource-consuming. On the other hand, exploratory analysis driven by computational techniques can alleviate this burden by narrowing the search scope, but the results are often deemed low-quality or non-specific by biologists. In this paper, we introduce a data-driven, statistics-based strategy to predict novel functional associations for transcription factors in the model plant *Arabidopsis thaliana*. To achieve this, we leverage one of the largest available gene expression compendia to build a genome-wide transcriptional regulatory network and infer regulatory relationships among transcription factors and their targets. We then use this network to build a pool of likely downstream targets for each transcription factor and query each target pool for functionally enriched gene ontology terms. The results exhibited sufficient statistical significance to annotate most of the transcription factors in Arabidopsis with highly specific biological processes. We also perform DNA binding motif discovery for transcription factors based on their target pool. We show that the predicted functions and motifs strongly agree with curated databases constructed from experimental evidence. In addition, statistical analysis of the network revealed interesting patterns and connections between network topology and system-level transcriptional regulation properties. We believe that the methods demonstrated in this work can be extended to other species to improve the annotation of transcription factors and understand transcriptional regulation on a system level.

## 1. Introduction

Transcription factors (TFs) are part of the cellular mechanisms that dictate variations in protein synthesis rate. They are proteins themselves that recognize and bind to distinct signals encoded in the DNA sequence of the genes and are often part of the transcription machinery. Aside from their prominent role in recognizing and binding to patterns in the gene, also known as binding motifs, it can be argued that TFs play a crucial role in biological functions. These functions can be enhanced or repressed by manipulating the transcription of the target genes that encode proteins responsible for performing the function. From this perspective, TFs can be viewed as “knobs” that control a set of output signals representative of the transcriptome. This makes transcription factors particularly important in the context of disease and drug discovery. However, the correct identification of the functions of a TF requires knowledge of its binding targets. Furthermore, transcription factors themselves, their binding motifs, and downstream targets can vary between species as well as tissues of the same organism. This fact further complicates the identification of the downstream targets of TFs, and by extension, their roles in modulating cellular functions. Another challenge lies in the identification of the binding motifs. Motif sequences and their lengths can vary between transcription factors and different species, though they are short in length (typically 5 to 15 base pairs). Experimental methods of binding-motif discovery can be challenging due to many different factors [1]. On the other hand, computational techniques [2,3,4,5] have to be utilized to scan DNA sequences of genes and identify short, conserved strings that appear in multiple genes. Candidate motifs can be used to guide practical motif discovery.

Transcriptional regulatory networks (TRNs), also called gene regulatory networks, are an effective tool for studying the complex relationships among TFs and their target genes. They are essentially graph-based representations of gene regulation. TRNs can be constructed based on curated experimental evidence of TF interaction and binding. A widely popular experimental technique for investigating TF binding locations is ChIP-seq. Using this method, the entire genome is probed for binding evidence. Nonetheless, our understanding of transcriptional regulation in many species remains incomplete. ChIP-seq depends on the availability of specific antibodies for TFs, is sensitive to stochastic effects, and can produce noisy results. On the other hand, computationally constructed TRNs can leverage the growing repository of expression profile datasets spanning a wide variety of treatment conditions. Many studies have delved into the data-driven analysis of experimental and simulated expression profiles as well as in silico regulatory network construction [6,7,8,9,10,11,12,13,14]. These networks prove to be particularly useful in identifying master regulators and gene modules and assigning functional roles. Computationally constructed TRNs can produce false-positive, and false-negative results [15] like other classification and inference techniques. Nonetheless, their importance can not be overstated as an orthogonal tool with respect to binding evidence, and they can highlight statistically significant regulatory interactions for experimental probing.

Numerous studies have been devoted to constructing and analyzing TRNs for the model plant *Arabidopsis thaliana*, most of which focus on specific gene/TF families and the biological processes that they regulate [16,17,18,19,20,21,22]. Several genome-scale TRNs have been computationally constructed by integrating multiple sources of data, such as promoter sequences, binding motifs, sequence conservation, and gene expression data [14,23,24]. However, binding site information is static and may not be available for all TFs/genes, while sequence conservation-based methods cannot detect regulatory relationships that have diverged in evolution. In this study, we aim to construct a genome-wide TRN for Arabidopsis based solely on gene expression data, and focus on an important but relatively neglected downstream application of computationally predicted TRN, which is to enhance the annotation of transcription factors, including the biological processes that they regulate and the binding sites that they recognize. To this end, we use a large-scale expression dataset alongside a comprehensive list of transcription factors to build our network. This network is then used to infer the target pool of each TF. We then analyze each target pool to find their representative biological functions, which can then be assigned to the TFs. The predicted functions for each TF are cross-referenced with currently known functions from a reference database, and the comparison presents an intuitive metric to evaluate the quality of the predictions. We also perform the discovery of statistically significant motifs present in the target pool of each TF and compare them with reference binding motifs. To demonstrate the effectiveness of our method, we show that the biological functions and motifs inferred by this purely analytical approach are consistent with the current knowledge base. We claim that the analysis presented in this paper provides critical insights into previously undiscovered regulatory interactions, functional roles, and binding patterns, and paves the way for a broader understanding of plant biology. Our method can also be applied to other species in order to simultaneously evaluate the method itself and aid biological knowledge discovery.

## 2. Materials and Methods

### 2.1. Method Overview

The functional annotation of most TFs in *Arabidopsis thaliana* is limited to their ability to bind DNA sequences and regulate gene expression without the specific biological processes being regulated or DNA binding patterns recognized by the TF. To systematically improve the annotation of TFs, we sought to utilize the most abundantly available functional genomics data from gene expression microarrays to construct a genome-scale transcriptional regulatory network (TRN) and then use the TRN for functional annotation and motif discovery. Importantly, it is the *collection of edges* in the inferred TRN that is used as *statistical evidence* for enrichment analysis and systems-level knowledge discovery. Therefore, a critical decision is to carefully weigh the tradeoffs between the number of edges in the TRN and the quality of such edges to achieve sufficient coverage and statistical significance.

**Figure 1:**
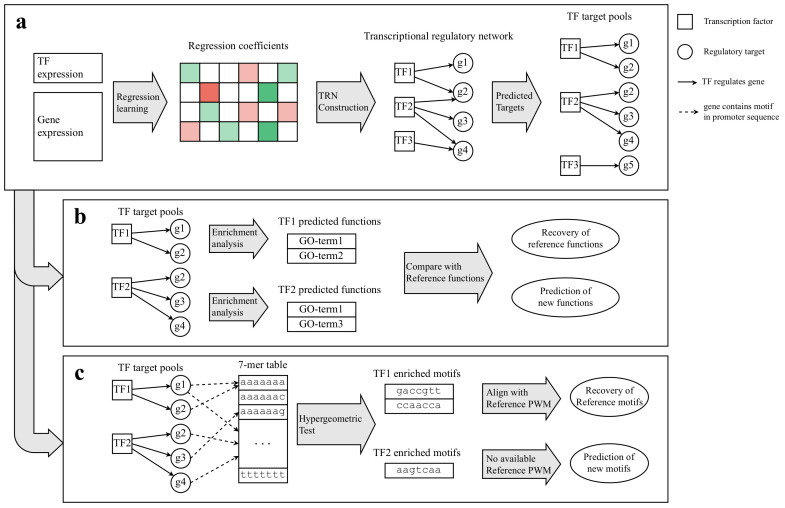
Overview of the methodology. (**a**) Gene expression data is fitted by regression models to produce the regression coefficients, which are then transformed into a transcriptional regulatory network. The predicted target genes for each transcription factor can be extracted from this network. (**b**) GO enrichment analysis on target pools yields statistically significant candidate functions for TFs. Candidate functions are cross-checked with the reference database. (**c**) Promoter sequences for genes associated with every 7-mer that is present in them. Hypergeometric test is used to identify statistically significant 7-mers enriched in the target pools of TFs. The significantly enriched 7-mers are compared with reference PWMs.

For computational simplicity and model robustness, we assume that the expression levels of a target gene can be approximated as a linear function of the expression levels of the TFs regulating that target gene. In reality, it is widely believed that TF–target interactions are nonlinear and context-sensitive. Linear approximation is a common practice in TRN inference [25] as it presents computational tractability and interpretability, and is adopted by numerous studies (e.g., [12,13,26]) as well as many participants in a community-wide assessment project [27]. On the flip side, more complex models may only result in a slight improvement in accuracy in gene expression modeling [27,28]. Under the linearity assumption, the task is reduced to a regression fitting problem, where feature sets are a set of TF expression levels, and dependent variables are the target gene expressions. We used the lasso regression model for the fitting since it penalizes a higher number of nonzero coefficients, thus eliminating weak regulatory associations. After fitting the observed data using regression, the fitted parameters can be used to construct the TRN, which then becomes the basis for inferring the target pool of each TF in the network and, consequently, for predicting candidate biological functions and binding motifs as well as systems-level analysis of network topologies (Figure 1).

### 2.2. Data Sources

The following datasets were used in this study:

- **Gene Expression:** Large-scale gene expression data [29] for *Arabidopsis thaliana*. The dataset contains genome-wide expressions from various treated/control samples collected at different time intervals from various tissue source sites, spanning approximately 7000 Affymetrix ATH1 profiles. Out of the 6057 treatment conditions, we used 3317, spanning four broad categories: abiotic stress, biotic stress, development, and hormone response.
- **TFs and associated motifs:** List of *Arabidopsis thaliana* transcription factors with a curated list of binding motifs from PlantTFDB [30]. Corresponding expression data were available for 1384 out of 1717 TFs.
- **TF functions:** Currently known biological functions associated with TFs from gene ontology [31].
- **Gene promoter sequences:** 25,516 promoter sequences associated with genes [32].
- **Gene loci:** Gene loci, lengths, and attributes acquired from TAIR [33].

### 2.3. Network Construction Using Lasso Regression

The regression model was fitted from the gene expression dataset using TF expressions as independent and target expressions as dependent variables. We define the following:

$$\begin{array}{cccc} & V & & :\;\mathrm{set}\;\mathrm{of}\;\mathrm{genes},\;|V|=n\\ & T & & :\;\mathrm{set}\;\mathrm{of}\;\mathrm{TFs}\;\left(\mathrm{features}\right)\;\mathrm{where}\;T\subseteq V,\;|T|=n_{T}\\ & m & & :\;\mathrm{number}\;\mathrm{of}\;\mathrm{treatment}\;\mathrm{conditions}\;\left(\mathrm{samples}\right)\\ & Y_{j}={\left\{y_{1},y_{2},\ldots ,y_{m}\right\}}^{⊤} & & :\mathrm{column}\;\mathrm{vector}\;\mathrm{of}\;\mathrm{expression}\;\mathrm{levels}\;\mathrm{of}\;\mathrm{gene}\;j\\ & X=\{Y_{i}:i\in T\} & & :(m\times n_{T})\;\mathrm{matrix}\;\mathrm{containing}\;\mathrm{expression}\;\mathrm{levels}\;\mathrm{of}\;\mathrm{TFs} \end{array}$$

The expression of gene *j* is modelled as $Y_{j}≃XA_{j}$ where $A_{j}={\left\{a_{1},a_{2},\ldots ,a_{n_{T}}\right\}}^{⊤}$ is the set of coefficients associated with each TF. Using regression estimate, we obtain $A_{j}$ for every gene *j*. The set of coefficients for the entire target gene list is represented as the $n_{T}\times n$ coefficient matrix, $A=\left\{a_{ij}\right\}=\left\{A_{j}:j\in V\right\}$, where $a_{ij}$ indicates the coefficient for TF *i* and gene *j*. As we will later discuss, this representation can be trivially transformed into a transcriptional regulatory network. We note that a model for each gene implies models for the independent variables, in other words, the expressions of the TFs as well. This is intentional, as a TF can itself be regulated by other TFs and master regulators. In practice, training a model for a TF *j* will most likely result in $a_{i}=0$ if i≠j and 1 otherwise. To circumvent this, we zeroed out the expression of TF *j* in the input samples when learning the model for the expression levels of *j*.

Prior to constructing the TRN, we focused on the fine-tuning aspects of the regression analysis. The value of the regularization parameter λ was tuned to maximize the mean $R^{2}$ of the regression models using 10-fold cross-validation, where an alternating 10 percent of samples were used for testing and the remaining 90 percent for training. Another step in the fine-tuning process involved removing genes that poorly fit into the regression model. In order to ensure the quality of the TRN, we removed target genes with mean $R^{2}$ values below 0.61 (25th percentile) in cross-validation, after which 15,273 target genes remained. We used this refined target gene list and all samples to train the final lasso regression model and learn the matrix *A*. *A* can be transformed into the weighted, directed regulatory network G=(V,E), where $E=\{\left(i,j\right):i\in T,j\in V,a_{ij}\neq 0\}$ is the set of regulatory edges. Edge weights are trivially defined as $w_{ij}=a_{ij}$, where $w_{ij}>0$ indicates up-regulation of gene *j* by TF *i* and $w_{ij}<0$ indicates down-regulation. For convenience, we also define the following terms:

(1) $$\begin{array}{cccc} {\mathrm{in}}_{j} & =\{i:(i,j)\in E\},\;\;\;\; & {\mathrm{out}}_{i} & =\{j:(i,j)\in E\}. \end{array}$$

Here, ${\mathrm{in}}_{j}$ refers to the set of TFs that have a regulatory edge with gene *j*, and ${\mathrm{out}}_{i}$ refers to the set of targets that have a regulatory edge from TF *i*. Before we applied this transformation from *A* to *G*, we performed one more refinement step. Although lasso regression minimizes the number of nonzero coefficients, the resulting model still predicted a high number of nonzero coefficients per TF. Therefore, to further reduce noise, we applied a cutoff α on the absolute value of the coefficients in *A* and acquired a new coefficient matrix $A^{*}$ as follows:

(2) $$A^{*}=\left\{a_{ij}^{*}\right\}\;\mathrm{where}\;a_{ij}^{*}=\left\{\begin{array}{cc} a_{ij}, & \mathrm{if}\;|a_{ij}|\geq \alpha \\ 0, & \mathrm{otherwise} \end{array}\right.$$

Finally, we applied the transformation described above to acquire *G* from $A^{*}$. from m=3317 samples. Additionally, we constructed treatment-specific GRNs for 1810, 519, 738, and 250 samples broadly categorized into four non-overlapping treatment types: abiotic stress, biotic stress, development, and hormone response, respectively. In subsequent discussions, the treatment-specific GRNs are referred to as $G_{\mathrm{treatment}}$.

### 2.4. Network Analysis

We inspected properties of *G*, such as clustering coefficient [34], degree coefficient [35], and network diameter, using the NetworkX python package [36]. In addition, the relationships between the dispersion of expression (defined in Equation (3)), mean expression, and gene length were established from the gene expression dataset *Y*.

(3) $$\begin{array}{cc} {\mathrm{Dispersion}}_{i} & =\frac{\mathrm{std}(Y_{i})}{\sqrt{\mathrm{mean}(Y_{i})}} \end{array}$$

### 2.5. Biological Function Enrichment Analysis

The inferred target pools for the transcription factors allow us to peer into their global as well as condition-specific functional roles. The intuition behind this approach is that if TF *i*’s putative target pool, ${\mathrm{out}}_{i}$ is significantly enriched in a set of biological functions, then TF *i* is likely involved in them as well. We used the DAVID tool [37] to run over-representation analysis on ${\mathrm{out}}_{i}$, the list of target genes for each TF *i*, where the statistical significance (the so-called EASE score) is computed using a modified and more conservative Fisher’s exact test [38]. We focused on only the biological process branch of Gene Ontology (GO) terms and used the default parameters unless otherwise specified. The analysis produced a set of enriched gene ontology (GO) biological process terms for each ${\mathrm{out}}_{i}$. The statistically significant annotations (EASE score ≤ 0.05) were then used subsequently for additional statistical analysis and annotations of the corresponding TF *i*. To address the potential multiple hypothesis testing problem, we adopted a random-sampling-based approach (see Section 2.5.1), which does not assume independence of the hypotheses and has been found to be more effective than other multiple testing correction methods for GO enrichment analysis [39,40,41]. As we will show in the results, this default threshold resulted in very few statistically significantly enriched terms for random gene sets and therefore is deemed sufficient for genome-scale annotations.

#### 2.5.1. Evaluating the Significance of the Number of Annotations

To assess the utility of TRN-based functional annotations, we first tested whether the majority of target pools we acquired from *G* are enriched in many more statistically significant GO terms than what would be expected of random gene sets of the same size. Even with multiple testing corrections, a gene set composed of random genes can still be enriched in some annotations by pure chance due to, for example, unrealistic assumptions made by the statistical test. However, a gene set with functionally similar members is likely to produce more statistically significant annotations than random gene sets, and we can expect this difference to be amplified over observations from many pairwise comparisons of network-derived and random gene sets.

Let, for transcription factor *i*, $|{\mathrm{out}}_{i}|=k_{i}$, and the number of enriched terms is $t(k_{i})$. We can randomly sample $k_{i}$ genes and perform enrichment analysis to obtain $t_{\mathrm{rand}}\left(k_{i}\right)$ terms. Repeating the random sampling 100 times, we can obtain the mean and standard deviation of $t_{\mathrm{rand}}\left(k_{i}\right)$, denoted as $\mu_{k_{i}}$ and $\sigma_{k_{i}}$. We now calculate the z-score for the number of significantly enriched terms from the target pool of TF *i* as follows:

(4) $$z_{i}=\frac{t\left(k_{i}\right)-\mu_{k_{i}}}{\sigma_{k_{i}}}$$

A high *z*-score indicates a greater deviation from the mean number of significant annotations enriched in random gene sets of the same size, which in turn suggests that the edges incident to TF *i* are more likely to be rooted in biological significance.

Note that $\mu_{k_{i}}$ and $\sigma_{k_{i}}$ only rely on the value of $k_{i}$, i.e., the target pool size, rather than the actual genes in the pool. Therefore the values can be estimated independent of the TFs for all possible target pool sizes. In practice, instead of performing enrichment analysis for random gene sets for all sizes, we only ran enrichment analysis on a small number of target pool sizes and fitted the values of $\mu_{k}$ and $\sigma_{k}$ as linear functions to *k*. The values of $\mu_{k_{i}}$ and $\sigma_{k_{i}}$ for each target set are then estimated with the linear functions given the target set size. Specifically, we randomly selected $k=\left\lfloor 2^{1.5}\right\rfloor ,\left\lfloor 2^{2}\right\rfloor ,\left\lfloor 2^{2.5}\right\rfloor ,\ldots \left\lfloor 2^{10}\right\rfloor$ target genes and performed GO-term enrichment on each of the randomly populated target pools. The experiment was repeated 100 times, and the observed $\mu_{k}$ and $\sigma_{k}$ values were used to construct linear regression models for estimating $\mu_{k_{i}}$ and $\sigma_{k_{i}}$ for any given $k_{i}$.

#### 2.5.2. Comparison of Functional Annotations to Known Annotations

In order to determine the degree to which the predicted functions agree with the current knowledge base, we applied a few strategies. First, we assessed the simple overlap between the predicted GO terms and a reference list of GO terms from TAIR November 2022 release [33]. Second, to measure the similarity between a predicted and reference GO term when they are not an exact match, we used GOSemSim [42]. Third, we also investigated whether the TRN-based annotations were able to improve upon the current knowledge base by annotating the TFs with more specific functional roles. Here, specificity is a trait of the GO terms themselves. Specificity for a term can be measured either as the shortest distance from the root of the GO term hierarchy graph or as the number of genes that are annotated with the term. In this study, we adopt the latter approach. Given a species and a GO term, the number of genes from this species that are associated with the GO term can be viewed as a metric of “generality” for that term. Terms that denote broad functional categories, such as biological process (GO:0008150) and response to stress (GO:0006950), are annotated to many genes in the species, while terms associated with more specific functional activities, such as cellular response to UV-B (GO:0071493) and root cap development (GO:0048829), are annotated to fewer genes. We define specificity, *c*, for individual GO terms as well as a set of terms as follows:

(5) $$\begin{array}{cc} \mathrm{specificity}(\mathrm{term}) & =\frac{1}{\#\mathrm{of}\mathrm{genes}\mathrm{annotated}\mathrm{with}\mathrm{term}} \end{array}$$

(6) $$\begin{array}{cc} \mathrm{specificity}({\mathrm{term}}_{1},{\mathrm{term}}_{2},\ldots ) & =\max(\mathrm{specificity}\left({\mathrm{term}}_{1}\right),\mathrm{specificity}\left({\mathrm{term}}_{2}\right),\ldots ) \end{array}$$

Equations (5) and (6) provide a straightforward approach to determining the specificity of the predicted annotation for a TF in our network. For each TF annotated with multiple terms, we consider the term with the highest specificity to be a metric of the overall specificity of the TF’s annotation.

#### 2.5.3. Predicting Novel Functional Annotations for Transcription Factors

There are many *Arabidopsis thaliana* TFs whose functions are not entirely understood [43]. The downstream enrichment analysis of TFs and their targets retrieved from our model can be used to suggest candidate functional annotations for many of them. While a more stringent *p*-value cut-off can be used to prioritize annotation terms that have greater statistical significance, we reported the finding with the default *p*-value threshold of 0.05 in the results to cover more TFs, alongside the supporting literature that corroborates some of the predicted functions but had not been included in reference databases at the time of conducting this study.

### 2.6. Motif Discovery and Alignment with Reference PWMs

We conducted an over-representation analysis and acquired candidate motifs that exhibit statistically significant enrichment in the promoter regions of TF target pools. We enumerated all *k*-mers with *k* = 6, 7, and 8 ($4^{6}+4^{7}+4^{8}$ = 86,016 total) and recorded the presence of each motif in the promoter region of the target genes. Our decision to use these *k*-mer lengths is informed by several factors. First, given the median promoter sequence length of 1577 bp, a smaller motif length would result in many matches simply by chance. On the other hand, most binding motifs have a relatively short conserved core, and a larger motif length (with no mismatch allowed) may significantly reduce the sensitivity of motif discovery. While it is possible to increase the length of the candidate motif by allowing mismatches, the strategy will significantly increase the running time given the number of TFs we are annotating, and properly evaluating the predictions will become more challenging. We also note that some post-processing strategies can be developed to refine the predicted binding motifs. In particular, longer binding sites can be represented as concatenations of multiple shorter motifs, while shorter binding sites can be represented as a collection of multiple motifs with high degeneracy in some positions. We decided not to perform any post-processing in this study for simplicity.

For a candidate motif *f* and TF *i*, let *L* denote the total number of genes that contain *f* in their promoter region, and *ℓ* denote the number of genes in ${\mathrm{out}}_{i}$ that contain motif *f*. We denote $|{\mathrm{out}}_{i}|=k_{i}$ and the total number of genes as *n*. The hypergeometric test (Equation (7)) was used to measure the statistical significance of the enrichment of motif *f* in the targets of TF *i*. Additionally, the *p*-values were corrected with a false discovery rate of 5% using the Benjamini–Hochberg method [44].

(7) p−value(fenrichedinouti)=∑ℓkiLℓn−Lki−ℓnki

For evaluation, we cross-matched the statistically significant 7-mers with the reference motifs available in the PlantTFDB database. The PlantTFDB dataset contains binding motifs represented as position weight matrices (PWMs) for 196 TFs. While our analysis was restricted to motifs with an exact length of 7 bp, the reference dataset contained motifs of varying lengths, most of which are longer. To check whether the computationally enriched motifs partially overlapped with PlantTFDB motifs, we scored each motif enriched in ${\mathrm{out}}_{i}$ against the corresponding PWM for TF *i*. The scoring scheme is rooted in the information content of a PWM. We denote a PWM, *p*, as a 4×t matrix where *t* is the length of the PWM and $p_{c}^{k}$ denotes the frequency of the *k*-th nucleotide symbol in the *c*-th column. We also define the column vector for the *c*-th column in the PWM as $p_{c}$, and a partial PWM through columns $c_{1}$ and $c_{2}$ as $p_{c_{1}\ldots c_{2}}$ where $c_{1}$ < $c_{2}$. The background frequency of a symbol *k* is denoted as $b_{k}$. We now define the following:

$$\begin{array}{cc} s(p_{c}) & =\max_{k=1}^{4}\left(-p_{c}^{k}\log\frac{p_{c}^{k}}{b_{k}}\right)\\ s(p_{c_{1}\ldots c_{2}}) & =s\left(p_{c_{1}}\right)+s\left(p_{c_{1}+1}\right)+\ldots +s\left(p_{c_{2}}\right)\\ s_{\max}\left(p,7\right) & =\max_{c=1}^{t-6}\;s\left(p_{c\ldots c+6}\right) \end{array}$$

We can also express a motif of length 7 as a 4×7 matrix *q*, where $q_{c}^{k}\in \left\{0,1\right\}$. If motif *q* is aligned to the partial PWM $p_{c\ldots c+7}$, then the resulting score is defined as $s(p_{c\ldots c+7}⊙q)$, where ⊙ indicates element-wise multiplication of the two matrices. Finally, we calculate the normalized maximum possible alignment score that can be acquired by aligning motif *q* to any starting position of the PWM *p*, as follows:

(8) $$\begin{array}{c} \mathrm{ic}-\mathrm{score}\left(p,q\right)=\max_{c=1}^{t-6}\frac{s(p_{c\ldots c+6}⊙q)}{s_{\max}\left(p,7\right)} \end{array}$$

## 3. Results and Discussion

### 3.1. Network Construction Process and Topological Properties

**Figure 2:**
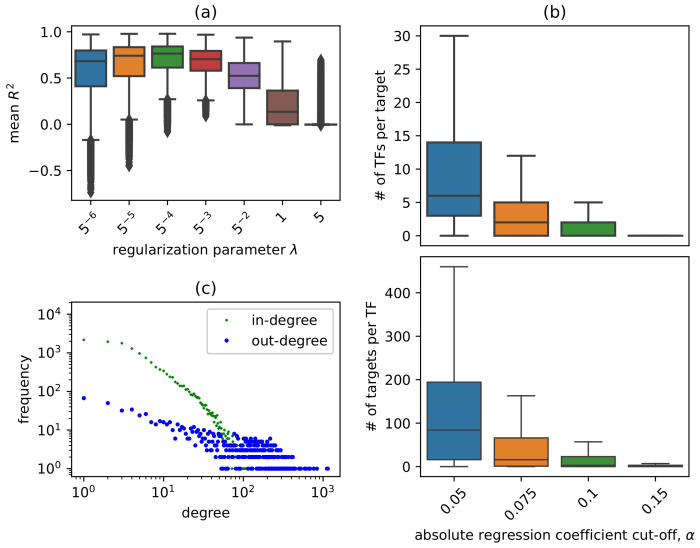
Parameter tuning and the degree distribution of the resultant network. (**a**) Distribution of mean $R^{2}$ from 10-fold cross-validation using a range of values for the regularization parameter λ. (**b**) Distribution of the number of predicted regulators, $|{\mathrm{in}}_{j}|$ (top panel), and number of predicted targets, $|{\mathrm{out}}_{i}|$ (bottom panel), with respect to the cut-off value for regression coefficient. Data points outside of two times inter-quantile range are excluded from the boxplot for clarity. Genes with no TFs predicted and TFs with no targets, collectively called singletons, are included in the boxplot. (**c**) Degree distribution of the resultant transcriptional regulatory network.

It is worth discussing the parameter tuning process before diving into the biological knowledge discovery implications of the TRN we constructed. To tune the L1 regularization parameter λ for lasso regression, we first fitted a set of models with a range of λ values. For each value of λ, we recorded the mean $R^{2}$ value for every gene after performing 10-fold cross-validation. Figure 2a shows the distribution of mean $R^{2}$ for each selection of λ, with candidate λ values ranging from $5^{-6}$ to 5 in geometric increments. The final selection for λ=0.008 was made based on the highest observed mean of the $R^{2}$ distributions. Figure 2b shows the distribution of $|{\mathrm{in}}_{j}|$ and $|{\mathrm{out}}_{i}|$ (defined in Equation (1)) for a range of α values. Note that data points outside of two times inter-quantile range are excluded from the boxplots for clarity. In addition, singletons (TFs without predicted targets and genes without predicted TFs) are included here for overall density consideration but are excluded from the final network for subsequent analysis. The coefficient cut-off parameter, α=0.075, for the final network, *G*, was empirically chosen to achieve a network with moderate density: in the resultant network, most target genes are regulated by a relatively small number of TFs ($|{\mathrm{in}}_{j}|\leq 13$), and most TFs regulate only a handful of genes ($|{\mathrm{out}}_{i}|\leq 180$). The coefficient matrix for constructing the GRN can be found in Supplementary Table S1. We found that the enrichment results acquired from *G* are not too sensitive to the choice of α. In addition to the global GRN, the context-specific GRNs for abiotic stress, biotic stress, development, and hormone were constructed using co-efficient cut-off values 0.08, 0.089, 0.095, and 0.078, respectively, in order to achieve network density similar to *G*, which facilitates downstream comparison.

Figure 2c shows that both the in-degree and out-degree of nodes in *G* roughly follow a power law degree distribution, consistent with the pattern frequently observed in biological networks. In addition, as shown in Table 1, the mean and median in-degree, or the number of regulators for each target gene, is 8.54 and 4, respectively, while the mean and median out-degree, or the number of target genes for each TF, is 97.26 and 54, respectively. The phenomenon that the mean degree is significantly larger than the median degree for both TF and targets and the small network diameter of 6 suggests that the transcriptional network has the property of a typical scale-free network. On the other hand, the relatively small clustering coefficient of 0.04 is a result of the primarily bipartite nature of the TRN, as most edges are between TFs and targets, with only a small fraction of edges between two TFs.

An interesting property of the TRN *G* is its negative degree coefficient of −0.24. This metric is calculated from the Pearson correlation between the degrees of the adjacent nodes of edges; for most edges, one end is a TF, and the other end is a target gene. (As mentioned above, there are only a small number of TF-TF edges). Therefore, the negative correlation implies that the network exhibits degree disassortativity: high-degree TF nodes tend to connect to low-degree target nodes, and low-degree TFs tend to connect to high-degree target nodes [45]. A possible interpretation from a systems perspective is that high-degree hub TFs that perform broad regulatory functions tend to regulate many low-degree targets. These low-degree target genes may have relatively simple functions, such as housekeeping genes whose expression level tends to be relatively stable. On the other hand, low-degree TFs usually regulate more specialized biological pathways that tend to consist of high-degree targets with diverse functions and complex expression patterns. Subsequent analysis involving gene expression data and gene length further confirms this hypothesis, as will be shown next.

### 3.2. Network Analysis Results Reveal Interesting Relationships among Gene Length, Gene Expression Patterns, and Gene Regulatory Complexity

Prior studies have established associations between gene architecture (e.g., gene length, codon bias, intergenic lengths, and intron length) and gene expression patterns and have suggested interesting evolutionary mechanisms [46,47,48,49,50]. For example, it was shown that in plants, highly expressed genes tend to be the longest, while it is the opposite in animals [51]. On the other hand, genes with longer upstream/downstream intergenic regions usually have lower expression levels but with more variability [48], suggesting that these genes are under greater regulatory control. However, the latter is hard to test without a quality genome-scale transcriptional regulatory network.

**Figure 3:**
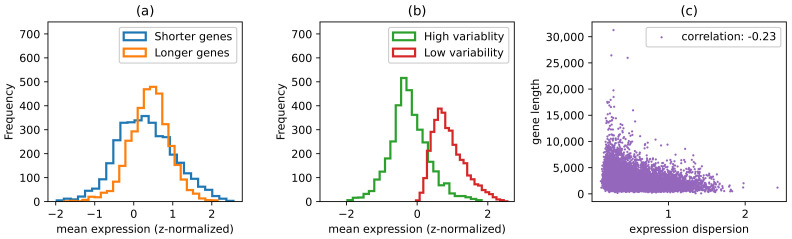
(**a**) Mean expression levels of genes with length above 75th percentile (long genes), and genes with length below 25th percentile (short genes). Long genes are highly expressed compared to short genes. (**b**) Mean expression levels of genes with expression dispersion above 75th percentile, and genes with dispersion below 25th percentile. Low dispersion genes are highly expressed compared to high dispersion genes. (**c**) Gene length vs. expression dispersion.

**Figure 4:**
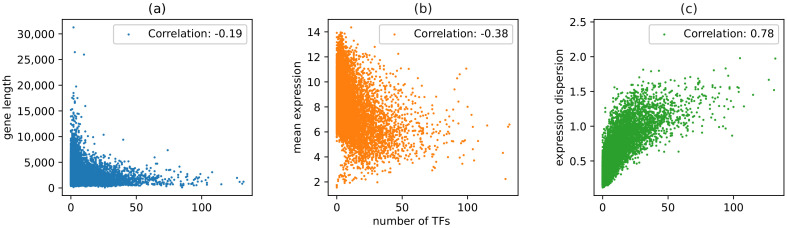
Correlation among gene length, number of TF, average expression, and their dispersion of expression. (**a**) Gene length negatively correlates with the number of predicted regulators, $|{\mathrm{in}}_{j}|$. (**b**) Mean expression has a negative correlation with $|{\mathrm{in}}_{j}|$. (**c**) Expression dispersion and $|{\mathrm{in}}_{j}|$ positively correlate.

As our computationally predicted transcriptional regulatory network included a large number of transcription factors and targets, we systematically evaluated the relationship among gene regulatory complexity, gene length, and gene expression variability and compared it with the existing results. To this end, we first computed the gene expression index of dispersion (see Methods) for each gene as an indication of its expression variability. Consistent with previous results [48,51], we found that highly expressed genes are more likely to be long and have low dispersion of expression, as shown in Figure 3a,b. It also follows that gene length is negatively correlated with expression dispersion (Figure 3c). Interestingly, as shown in Figure 4, gene length has a negative correlation with the number of TFs operating on the gene ($|{\mathrm{in}}_{j}|$) with a Pearson correlation coefficient of −0.19 (*p*-value $=2\times 10^{-124}$). Furthermore, $|{\mathrm{in}}_{j}|$ has a negative correlation with mean expression (Pearson correlation coefficient: −0.38, *p*-value =0.0), as shown in Figure 4b. This suggests that genes regulated by a high number of TFs tend to be short and generally have lower expression levels for faster response to diverse conditions. Indeed, $|{\mathrm{in}}_{j}|$ is strongly positively correlated with the dispersion of expression (Figure 4c), confirming that those with more regulators have more complex expression patterns. To verify our results, we also analyzed a dataset that contains computationally predicted TFBS for 400 TFs in a genome scale [24]. We observed that the number of unique TFBS in the promoter of each gene has a negative correlation with gene length (Supplementary Figure S1, Pearson coefficient = −0.045, *p*-value = $1.8\times 10^{-8}$), which is consistent with but not as strong as the results obtained from our predicted networks, likely due to the relative incompleteness of their dataset with fewer TFs and a requirement for the TFBS to be conserved in multiple species as part of their prediction algorithm.

Taken together, our network analysis results confirmed that in Arabidopsis, longer genes are more likely to be housekeeping genes that exhibit stable and high expression levels and require fewer regulators, while shorter genes generally have more complex but lower expression with a greater need for regulation with respect to internal/external condition changes. Although indirect, these results suggest that our regression-based method for constructing a transcription regulatory network is able to capture, at least on a high level, the structure of the regulatory relationships among a large number of TFs and their target genes, which led us to explore the possibility of network-based annotation of transcription factors.

### 3.3. Biological Function Enrichment Analysis Results

#### 3.3.1. Target Pools of TFs Are Enriched in a Significant Number of Functional Terms

**Figure 5:**
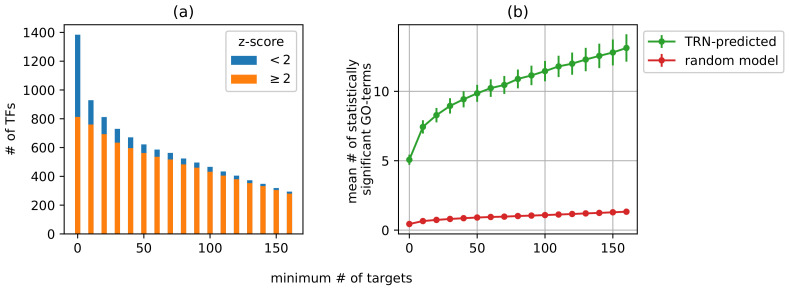
TRN-derived TF targets are enriched in a relatively higher number of GO terms. (**a**) Most of the transcription factors are enriched in more annotations compared to random target sets, and the percentage of highly annotated TFs grows with the minimum target count cutoff. (**b**) Shows a 95% confidence interval of the mean number of significant GO terms.

Using the target pool ${\mathrm{out}}_{i}$ as input gene sets for functional enrichment analysis, we were able to annotate most of the corresponding transcription factors in *Arabidopsis thaliana*. As we will later discuss, the predicted annotations confirm and contribute to the curated reference annotations. To begin with, we focus on the number of TFs that were annotated and the number of annotations. The number of annotations for the target pool of each transcription factor was compared to the expected number of annotations for a random target pool of the same size using a *z*-score (see Section 2.5.1). If $z_{i}\geq 2$, we consider the number of GO terms reported for ${\mathrm{out}}_{i}$ to be significantly higher than what would be expected of a random selection of genes. Among all TFs, 59% had $z_{i}\geq 2$. This percentage is higher if TFs with fewer targets are ignored. For example, 90% of the TFs with no fewer than 50 targets had $z_{i}\geq 2$ (Figure 5a). Additionally, we compared the mean number of significant GO terms between *G* and the random target model. Collectively, the TRN-derived target pools have a consistently greater mean number of significant annotations compared to random target pools. For example, the predicted target genes sets of transcription factors with no fewer than 50 targets are enriched in 10 GO terms on average, whereas random target sets with the same cardinality are enriched in <1 GO term on average (Figure 5b). In addition, these results are not sensitive to the network construction parameters (Supplementary Figure S2). This enforces our confidence in the biological significance of the constructed transcriptional regulatory network.

#### 3.3.2. Predicted Functional Annotations Reflect and Improve Reference Annotations

**Figure 6:**
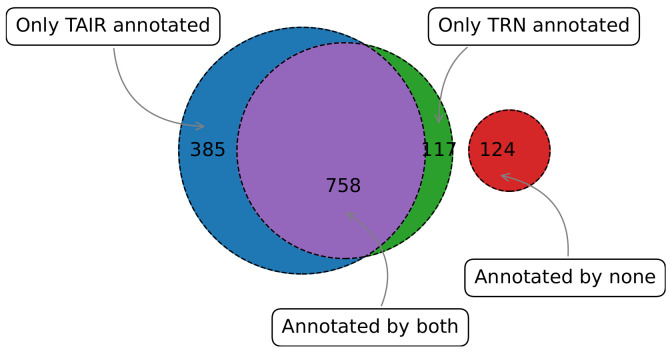
TF function assignment capacity of the TRN compared to the reference database of annotations.

Using our method, we were able to successfully annotate a large number of TFs that had two or more genes in the target pool with at least one functional term. Functional annotations were generated for TFs with existing annotations as well as TFs for which there are no known functions in the reference database [33]. Figure 6 shows the number of annotated TFs, with over half of the TFs being annotated by both our method and the reference. From the 1384 transcription factors in *G*, $|{\mathrm{out}}_{i}|>2$ in 82%, whereas 875 (≃63%) TFs were enriched in at least one GO term. The 117 TFs marked as having no functions in the reference database are actually trivially annotated (i.e., regulation of transcription); therefore, we ignored these annotations.

**Figure 7:**
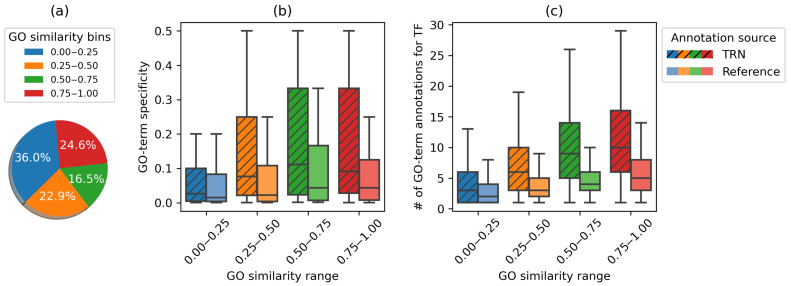
(**a**) Similarity between reference and predicted functional annotations. TFs are binned based on the similarity score between the reference and predicted GO terms. (**b**) Distribution of TF annotation specificity (Equation (6)) for GO terms predicted by TRN or listed in the reference database. TRN-predicted GO terms have higher specificity. (**c**) Distribution of the number of GO terms for individual TFs predicted by TRN and available in the reference database. TRN is able to predict more functional annotations than are listed in the current knowledge base.

There is considerable agreement between the curated reference annotations and the annotations we derived using the computational method. We observed that 153 (∼13%) TFs have at least one predicted biological process annotation in common with the reference annotations. To further investigate the degree to which our TRN-predicted annotations agreed with reference annotations, we used GOSemSim [42]. We computed Wang’s graph-based semantic similarity [52] for the 758 TFs that had at least one annotated term from TRN and the reference database. The similarity score ranges between 0 and 1, and we divided the TFs into four bins depending on their similarity score. The similarity score suggests agreement between reference GO annotations and TRN-derived GO annotations, with over one-third of the TFs having a similarity score ≥0.5 (Figure 7a).

TRN-based enrichment analysis can assign more specific candidate functional roles to TFs with existing annotations in the reference database. Given two different sets of functional annotations for an individual TF acquired from TRN and the reference database, we can compare their specificity (Equation (6)). TRN predicts more specific GO terms for TFs (Figure 7b), making it possible to annotate TFs with more informative functional roles.

#### 3.3.3. TRN Predicts Reliable Novel Functional Annotations

The presently available TF functional annotations for *Arabidopsis thaliana* can be considered incomplete [53]. We propose that many of the GO term enrichments reported in our findings that are absent in the reference database can be considered candidate novel annotations for the transcription factors. Our method can generate additional candidate functional annotations for TFs that are trivially or partially annotated. Figure 7c shows the distribution of the number of reported GO terms by TRN against the number of reference GO terms (TAIR). Our analysis predicted more insightful annotations for 117 TFs that were trivially annotated in the reference database. Some of these findings corroborate functional annotations that are suggested by other studies but not included in the TAIR reference data source. For example, our analysis suggests that the transcription factor CDF3 (AT3G47500) is associated with circadian rhythm (GO:0007623), also reported in [54]. Similarly, the transcription factor AtIDD11(AT3G13810) may be associated with defense response to fungus (GO:0050832), also suggested by [55,56]. Several of these novel predicted functional annotations are reported in Table 2 with corresponding *p*-values and fold enrichment. The complete results can be found in Supplementary Table S2.

#### 3.3.4. TFs Exhibit Unique Condition-Specific Functional Roles

In addition to the annotations yielded from *G*, we also performed GO biological process enrichment analysis on the four treatment-specific GRNs, $G_{\mathrm{abiotic}-\mathrm{stress}}$, $G_{\mathrm{biotic}-\mathrm{stress}}$, $G_{\mathrm{hormone}}$, and $G_{\mathrm{development}}$. We observed that some TFs exhibited a preference for more functional annotations under a specific type of treatment. HD2C (AT5G03740) is associated with abiotic stress response related to water deprivation, whereas WRKY69 (AT3G58710) is associated with wounding [31]. The targets of HD2C and WRKY69 are enriched in GO-bp terms for GO:0009414 (response to water deprivation) and GO:0009611 (response to wounding), respectively, when the enrichment analysis is performed on $G_{\mathrm{abiotic}-\mathrm{stress}}$, but not for the global GRN. Similarly, AT1G42990 has been reported to be associated with pathogen response [57], and is enriched in GO:0042742 (defense response to bacterium) in $G_{\mathrm{biotic}-\mathrm{stress}}$ but not in *G*. The complete enrichment results can be found in Supplementary Tables S3–S6.

### 3.4. Motif Enrichment Recovers Reference PWMs and Identifies Candidate Binding Motifs

We identified significantly enriched *k*-mers from the pool of target genes regulated by each TF as the candidate binding motifs for the TF. Supplementary Tables S7–S9 contain the complete list of TF–motif pairs for all *k*-mers of lengths between 6 and 8. With an FDR ≤0.05 cutoff and a k=7, a total of 3645 TF–motif pairs were found to be statistically significant. Among these pairs, there are a total of 420 unique TFs and 1500 unique motifs. With k=6, we found more than 37000 TF–motif pairs that covered 791 unique TFs, while with k=8, only less than 100 TF–motif pairs were identified. To determine if the number of enriched TF–motif pairs bears any significance, we randomly rewired the GRN 100 times and compared the enrichment results for 7-mers. To rewire the GRN, the cardinality of ${\mathrm{out}}_{i}$ was unchanged for every TF *i*, while the members of ${\mathrm{out}}_{i}$ were randomly shuffled. We counted the number of random shuffles that produced at least as many significantly enriched TF–motif pairs as *G* did. Out of 100 runs, 97 runs resulted in no single TF–motif pair having an adjusted *p*-value ≤ 0.05. The other 3 runs yielded just one significant TF–motif pair, a considerable deviation from the thousands of statistically significant TF–motif pairs extracted from the TRN constructed using our method. This suggests that the network we built was able to reflect the underlying biology of transcription factor binding, as the alternative would result in a similar number of enriched motifs from the TRN-derived TF–motif associations and the randomly shuffled variant.

**Figure 8:**
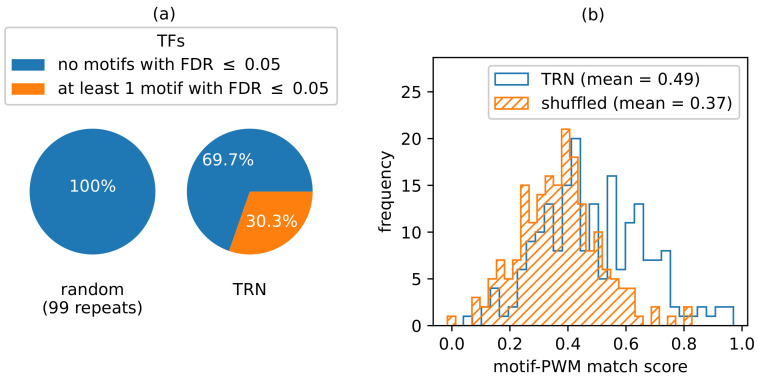
(**a**) Percentage of TFs with at least one significantly enriched motif from the motif enrichment analysis on the original TRN and a randomly shuffled variant. No motifs were enriched with FDR ≤0.05 in the randomly shuffled TRN. (**b**) Distribution of match scores with PlantTFDB reference PWMs for the respective transcription factors from all enriched motifs (no FDR cutoff applied) from the original and the randomly shuffled motifs.

**Figure 9:**
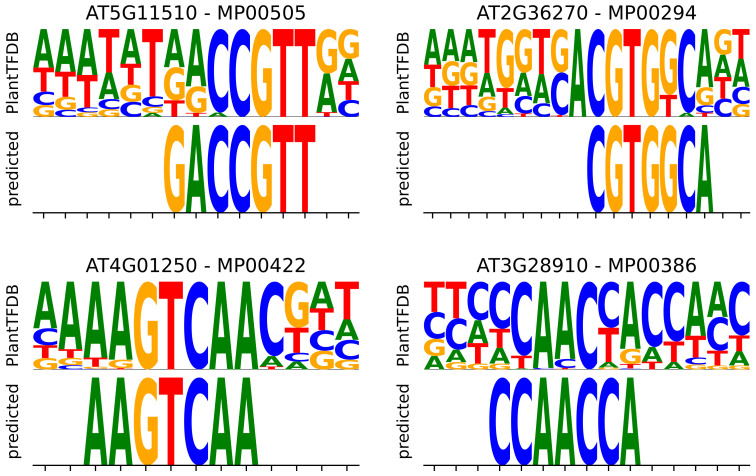
TRN-predicted motifs aligned to their best matching position in the respective PWM from the reference database. Top-scoring motifs are shown.

Comparing the significantly overrepresented motifs with the corresponding transcription factor motif PWMs from the reference database reveals that many of the computationally identified motifs are partial recoveries of the PWMs. We scored the enriched 7-mers against reference PWMs that were available using an information-content-based scoring scheme (Equation (8)). The motifs enriched in our analysis correspond to the highly conserved regions of the reference PWM. Again, to test whether these results can be acquired from random noise, we shuffled motifs among TFs and computed the scores of the shuffled motifs against PWMs. The distribution of the TRN motif scores is skewed towards the right (Figure 8b) compared to the shuffled motif score distribution, with an independent *t*-test *p*-value of $5\times 10^{-12}$. It should be noted that the PWMs were not part of candidate motif discovery and were only used for validation purposes. The differences in the matching score distribution between TRN-predicted and randomly selected motifs indicate that the TRN motifs are more likely to be aligned with ground-truth observations. Figure 9 shows some of the high-scoring motif logos aligned to the respective reference PWMs. We claim that the motifs predicted for TFs with no reference motifs can be considered candidate binding motifs and prioritized based on the adjusted *p*-value and fold change of enrichment.

## 4. Conclusions

In this paper, we proposed a network-oriented method for transcription factor functional annotation and binding site discovery. We applied this methodology to the model plant *Arabidopsis thaliana* and built a transcriptional regulatory network from genome-wide expression profiles. The network revealed an interesting association between the regulatory complexity and functional complexities of genes. In addition, functional enrichment analysis identified a statistically significant number of enriched functions for most of the TFs, many of which are novel or are more specific than the previously annotated functions. We also discovered possible binding sites for many TFs with statistical significance. The results presented here suggest that our network-oriented computational analysis can be effective in uncovering the regulatory relationships in *Arabidopsis*, which in turn can be utilized to annotate transcription factors with novel functional roles and DNA binding patterns. Our workflow makes use of the vast majority of gene expression data and is applicable to other species that are yet to be annotated.

## Figures and Tables

**Table 1:** Network statistics.

Network Attribute	Value
Number of nodes	14,043
Number of edges	117,587
Mean degree	16.75
Largest degree	1156
Number of TFs	1213
Number of targets	13,813
Mean (median) out-degree of TFs	97.26 (54)
Mean (median) in-degree of targets	8.54 (4)
Diameter ^1^	6
Clustering coefficient	0.04
Degree coefficient	−0.24

^1^ calculated for the undirected version of the network.

**Table 2:** Novel functional annotations for a few transcription factors.

Gene ID	Common Name	GO Term Description	*p*-Value	Benjamini	Fold Enrichment
		translation	$1.50\times 10^{-38}$	$1.30\times 10^{-35}$	4.22
AT2G37120	S1FA2	ribosome biogenesis	$1.18\times 10^{-24}$	$5.12\times 10^{-22}$	5.72
		cytoplasmic translation	$1.53\times 10^{-9}$	$4.41\times 10^{-7}$	6.32
		plant-type cell wall organization	$1.03\times 10^{-5}$	$3.31\times 10^{-3}$	8.38
AT2G28510	DOF2.1	hydrogen peroxide catabolic process	$9.91\times 10^{-5}$	$1.59\times 10^{-2}$	7.34
		response to oxidative stress	$5.43\times 10^{-4}$	$5.81\times 10^{-2}$	3.32
		photosynthesis	$5.39\times 10^{-25}$	$1.18\times 10^{-22}$	24.94
AT1G68520	BBX14	protein–chromophore linkage	$4.75\times 10^{-6}$	$3.45\times 10^{-4}$	23.33
		photosynthetic electron transport in photosystem I	$7.10\times 10^{-6}$	$3.87\times 10^{-4}$	37.59
		flavonoid biosynthetic process	$1.22\times 10^{-4}$	$1.95\times 10^{-2}$	5.99
AT3G47500	CDF3	cellular response to hypoxia	$1.46\times 10^{-4}$	$1.95\times 10^{-2}$	17.75
		response to karrikin	$5.93\times 10^{-4}$	$1.95\times 10^{-2}$	4.75
AT1G76580	F14G6.18	removal of superoxide radicals	$1.42\times 10^{-4}$	$4.02\times 10^{-2}$	36.52
		hydrogen peroxide catabolic process	$6.89\times 10^{-4}$	$1.08\times 10^{-1}$	6.49
AT3G21270	ADOF2	thalianol metabolic process	$8.31\times 10^{-4}$	$1.08\times 10^{-1}$	59.29
		response to toxic substance	$1.52\times 10^{-3}$	$1.08\times 10^{-1}$	7.26
		oxidation-reduction process	$1.14\times 10^{-5}$	$4.01\times 10^{-3}$	2.30
AT1G21450	F24J8.8	response to oxidative stress	$1.14\times 10^{-5}$	$4.15\times 10^{-2}$	3.63
		embryo development	$2.37\times 10^{-4}$	$8.27\times 10^{-2}$	64.26
		embryo development ending in seed dormancy	$7.59\times 10^{-11}$	$5.15\times 10^{-9}$	10.14
AT5G10120	EIL4	lipid storage	$4.68\times 10^{-7}$	$1.53\times 10^{-5}$	73.36
		seed oilbody biogenesis	$6.78\times 10^{-7}$	$1.53\times 10^{-5}$	187.80
AT1G28050	BBX13	circadian rhythm	$2.75\times 10^{-5}$	$7.07\times 10^{-3}$	8.99
		response to hydrogen peroxide	$1.45\times 10^{-3}$	$1.86\times 10^{-1}$	10.07
AT4G29190	AtC3H49	response to toxic substance	$8.08\times 10^{-6}$	$2.68\times 10^{-3}$	10.73
AT3G18400	NAC058	suberin biosynthetic process	$3.15\times 10^{-15}$	$1.86\times 10^{-13}$	191.72
		lipid catabolic process	$2.07\times 10^{-3}$	$6.11\times 10^{-2}$	15.14
AT2G23290	AtMYB70	plant-type cell wall organization	$8.76\times 10^{-5}$	$1.89\times 10^{-2}$	13.04
		removal of superoxide radicals	$3.22\times 10^{-5}$	$6.43\times 10^{-3}$	59.76
AT2G37650	F13M22.15	cellular response to high light intensity	$1.18\times 10^{-3}$	$1.18\times 10^{-1}$	56.02
		response to copper ion	$7.10\times 10^{-6}$	$1.13\times 10^{-1}$	37.59
AT2G40740	ATWRKY55	defense response	$1.27\times 10^{-3}$	$1.15\times 10^{-2}$	27.99
		embryo development ending in seed dormancy	$9.54\times 10^{-7}$	$2.73\times 10^{-4}$	4.03
AT5G52010	AT5G52010	protein refolding	$3.79\times 10^{-6}$	$5.44\times 10^{-4}$	40.97
		response to heat	$1.20\times 10^{-5}$	$1.15\times 10^{-3}$	6.10
AT5G05790	AT5G05790	hydrogen peroxide catabolic process	$9.14\times 10^{-5}$	$9.32\times 10^{-3}$	20.42
		response to oxidative stress	$1.97\times 10^{-4}$	$1.00\times 10^{-2}$	7.95
		photosynthesis	$2.85\times 10^{-11}$	$1.05\times 10^{-8}$	9.31
AT3G24490	AT3G24490	photosynthesis, light harvesting in photosystem I	$7.89\times 10^{-5}$	$1.31\times 10^{-2}$	20.34
		reductive pentose-phosphate cycle	$1.06\times 10^{-4}$	$1.31\times 10^{-2}$	18.98
AT1G32360	F27G20.10	response to bacterium	$1.51\times 10^{-4}$	$4.81\times 10^{-2}$	6.89
		amino acid transmembrane export	$6.54\times 10^{-7}$	$1.06\times 10^{-4}$	176.96
AT1G64620	DOF1.8	response to salicylic acid	$9.62\times 10^{-5}$	$7.80\times 10^{-3}$	9.24
		seed development	$2.75\times 10^{-2}$	$1.53\times 10^{-1}$	13.88
AT1G60240	AT1G60240	cell wall modification	$3.71\times 10^{-5}$	$1.15\times 10^{-4}$	210.42
		pectin catabolic process	$5.76\times 10^{-5}$	$1.15\times 10^{-4}$	169.16

## Data Availability

Code and associated data sets are available upon request from the corresponding author.

## Supplementary Materials

The following supporting information can be downloaded at: https://www.mdpi.com/article/10.3390/genes1010000/s1. Figure S1: Number of unique TF binding sites vs. gene length; Figure S2: Number of enriched GO terms using different coefficient cutoff values; Table S1: coefficient matrix for constructing GRN; Table S2: Predicted functional annotations for each TF from global GRN; Table S3: Predicted functional annotations for each TF from abiotic stress specific GRN; Table S4: Predicted functional annotations for each TF from biotic stress specific GRN; Table S5: Predicted functional annotations for each TF from development specific GRN; Table S6: Predicted functional annotations for each TF from hormone response specific GRN; Table S7: Predicted 6-mer binding motifs for each TF; Table S8: Predicted 7-mer binding motifs for each TF; Table S9: Predicted 8-mer binding motifs for each TF.

## Abbreviations

The following abbreviations are used in this manuscript:

FDR	False discovery rate
GO	Gene ontology
TRN	Transcriptional regulatory network
PWM	Position-weight matrix
TF	Transcription factor
