# Supplementary material for: A Network-Based Approach for Improving Annotation of Transcription Factor Functions and Binding Sites in Arabidopsis thaliana
Source: Genes (Basel). 2023 Jan 21;14(2):282. doi: 10.3390/genes14020282 (PMC9957447; doi:10.3390/genes14020282)
Supplement: Supplementary file 1 [file genes-14-00282-s001.zip › Figurea S2.pdf]

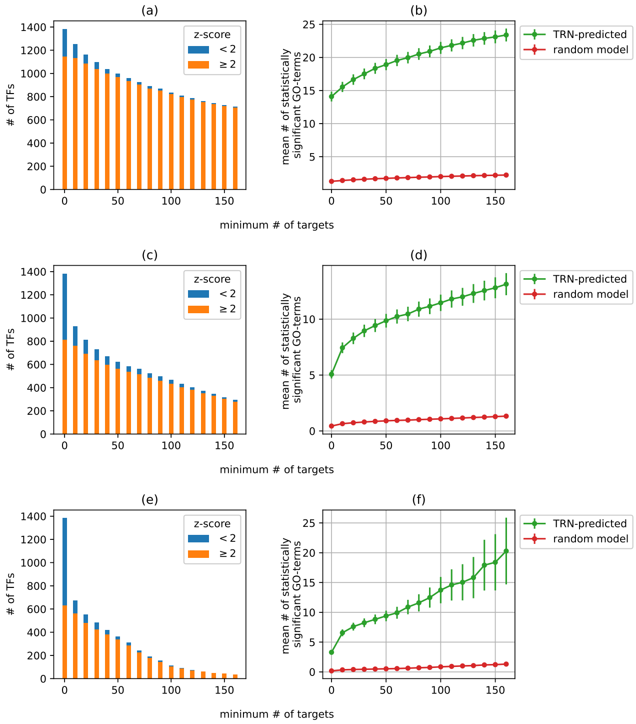

Figure S2: TRN-derived TF targets are enriched in a relatively higher number of GO-terms for the coefficient cut-off values 0.05 **(a-b)**, 0.075 **(c-d)**, and 0.1 **(e-f)**.
