# Supplementary figures and images for: A Network-Based Approach for Improving Annotation of Transcription Factor Functions and Binding Sites in Arabidopsis thaliana
Source: Genes (Basel). 2023 Jan 21;14(2):282. doi: 10.3390/genes14020282 (PMC9957447; doi:10.3390/genes14020282)

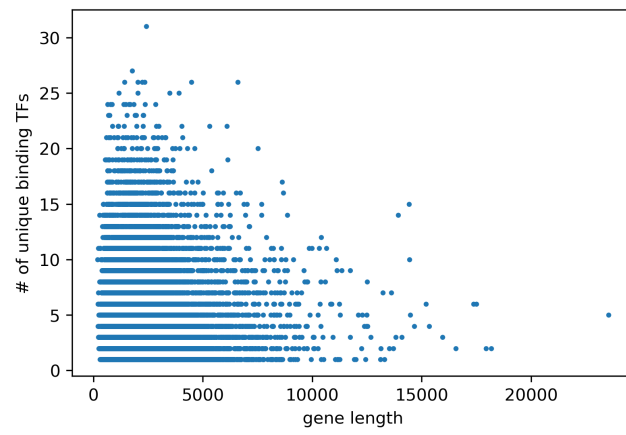

Figure S1: Number of unique TFBS vs gene length

Supplement: Supplementary file 1 [file genes-14-00282-s001.zip › Figurea S1.pdf]
